# Misleading terminology in pathology: lack of definitions hampers communication

**DOI:** 10.1007/s00428-021-03069-7

**Published:** 2021-04-02

**Authors:** Zi Long Chow, Blanca Iciar Indave, Menaka Dilani Samarawickrema Lokuhetty, Atsushi Ochiai, Ian A. Cree, Valerie A. White

**Affiliations:** 1 WHO/IARC Classification of Tumours, International Agency for Research on Cancer (IARC), 150 Cours Albert Thomas, 69372, CEDEX 08 Lyon, France; 2 School of Medicine, University of Tasmania, 41 Charles St, Launceston, TAS 7250 Australia; 3 National Cancer Centre, Kashiwa, Japan

**Keywords:** Medical terminology, Communication, Classification of tumors

## Abstract

Accurate terminology is the basis for clear communication among specialists and relies upon precise definitions, indispensable for the WHO Classification of Tumours. We identified a number of potentially misleading terms in use in the recently published WHO Classification of Tumours, 5^th^ edition. From a list of common sources that might be consulted by specialists in the pathology field, we searched for definitions of the terms. Where at least two sources provided definitions for a term, we assessed their level of agreement using an ad hoc developed scale. We identified 26 potentially misleading terms from the 5^th^ edition Digestive System and Breast Tumour Books, and 16 sources. The number of definitions provided by the sources ranged from no definition (for four terms) to ten (for two terms). No source had definitions for all terms. We found only 111 (27%) of a possible 416 definitions. Where two or more definitions were present for a term, the level of agreement between them was judged to be high. There was a paucity of definitions for potentially misleading terms in the sources consulted, but there was a good agreement when two or more definitions were present. In a globalized world where healthcare workers and learners in many fields may access these sources to learn about terminology with which they are unfamiliar, the lack of definitions is a hindrance to a precise understanding of classification in the speciality of pathology and to clear communication between specialist groups.

**Supplementary Information:**

The online version contains supplementary material available at 10.1007/s00428-021-03069-7.

## Introduction

The International Agency for Research on Cancer (IARC) is the specialized cancer research agency of the World Health Organization (WHO). A significant feature of IARC is the publication of the WHO Classification of Tumours (WCT), the knowledge base that underpins cancer diagnosis worldwide. This series of authoritative reference books, also known as the WHO Blue Books [1], is an important resource for pathologists, medical professionals, and cancer researchers globally, providing standards to support diagnosis, treatment, prognostication, and cancer research.

Terminology is the basis for specialist communication and should be used with precision in classifications. The complexity of technical content and of specialist knowledge, as well as the overlapping of specialties and fields, makes it increasingly relevant to pay attention to the accuracy of terminology especially across languages and geopolitical frontiers [2]. Misleading terminology has been recognized as an issue in all fields of technical writing, including the field of healthcare [3–10].

Definitions exist to describe a concept with precision. Three of the most authoritative dictionaries in the world provide similar definitions of the term “definition” as “constituting a precise statement of the essential nature or meaning of something” [11–13].

IARC aims to provide clear descriptions of tumors to aid in the correct diagnosis wherever these books are used. Each section describing a tumor begins with a concise definition of precisely what that tumor is before the entity is further delineated under pre-defined headings describing the different aspects that classify a tumor [1]. For patients, this means that their diagnosis is relevant and comparable worldwide and improvements from new research may be applicable for them. For researchers, precise terminologies mean that studies can be reproduced and compared with greater accuracy. Epidemiologists rely on clear definitions to monitor and compare tumors across different countries and registries.

As part of a quality improvement effort, we aimed to assess the extent of usage of potentially misleading terminology in the 5^th^ edition WHO Blue Books, by obtaining and comparing standard definitions for a selection of terms that we considered potentially misleading.

The specific objectives of this project were:

1. To identify a list of potentially misleading terms used in the recently published WHO Blue Books, 5^th^ edition.
2. To search for definitions of the selected terms in common official sources from the pathology and cancer domains.
3. To assess the extent of agreement between the sources.

## Methods

### Identification of misleading terms

A working group (WG) composed of three senior pathologists from the WCT group (VAW, IAC, MDSL) and a visiting senior pathologist (AO) identified by consensus a list of potentially misleading terms with reasons why they might be considered misleading (Table 1). The potentially misleading terms were divided into eight categories: one prefix, two types of suffixes, eponyms, latinate terms, and the three paired terms of microinvasion/microinvasive, dysplasia/dysplastic, degeneration/degenerative. For the prefixes and suffixes, we did a preliminary search in the two published 5^th^ edition Blue Books [14, 15] to look for the most common words with which these were associated and included these.

**Table 1:** List of potentially misleading terminologies identified

Potentially misleading terms	Why are they misleading, imprecise, or confusing?
Prefix (pseudo-)	
Pseudo-	Term used when something appears to be something else; imprecise
Pseudotumor	Term used to refer to any number of pathologies, both benign and malignant, that may produce a mass; imprecise
Pseudolymphoma	Old term used to refer to an inflammatory lesion that mimics a lymphoma, not any longer recommended; imprecise
Pseudoinvasion or pseudoinvasive	Spectrum of histologic changes producing the appearance of invasion; imprecise
Suffix (-oid)	
-oid	Suffix used to create an adjective; used when something is like something else; imprecise and undefined
Carcinoid	Term that is falling out of favor as there are more precise definitions of this neuroendocrine tumor; imprecise
Epithelioid	Adjective used to describe many different types of cells that look like epithelial cells to the microscopist, but for which the cell type is often not specified in a description; imprecise
Rhabdoid	Used in an undefined manner for a number of tumor appearances; imprecise
Pagetoid	Used to refer to a spectrum of appearances in an undefined manner; imprecise
Suffix (-like)	
-like	Similar to –oid, but by usage and convention is applied to different terms; undefined
Adenoma-like	Term used to mean something that is not a neoplasm, but simulates a benign epithelial neoplasm; undefined
Osteoclast-like	Used to refer to multi-nucleated giant cells that appear to look like osteoclasts, but are not situated in bone; undefined
Microinvasion/microinvasive	May be different definitions of this term depending on location; imprecise
Dysplasia	
Dysplasia	Two definitions of dysplasia; often used imprecisely
Serrated dysplasia	Often used in an undefined manner
High-grade dysplasia	Often used imprecisely
Low-grade dysplasia	Often used imprecisely
Dysplastic epithelium	Often used imprecisely
Degeneration/degenerative: what is degenerative? How do we know it is degenerative?	
Malignant degeneration	Lower grade neoplasms do not degenerate, they transform into high-grade neoplasms
Cystic degeneration	Most lesions do not truly form epithelial-lined cysts
Degenerative nuclear atypia	Imprecise array of nuclear changes that overlap with malignant changes; implies that a judgment has been made that a lesion is not malignant
Degenerative changes	Refers to a spectrum of changes that may or may not be ‘degenerative’
Eponyms: may be difficult to remember and often used in days before lesions were fully defined; many eponymous conditions have been shown to be other entities upon recent investigation and are therefore used improperly	
Barrett esophagus	Refers to a spectrum of changes occurring in lower esophagus that can be defined more precisely
Langerhans cell histiocytosis	Refers to a spectrum of disease processes that are now considered to be neoplasms
Paget disease	Two distinct types of Paget disease; term often used imprecisely when referring to intraepithelial lesions
Latinate terms: Difficult to use and remember	
Leiomyomatosis peritonealis disseminata	

### Sources of definitions

The WG agreed upon a list of sources to search for definitions of the selected terms. The sources included the WHO web page, the websites of several pathology organizations, medical dictionaries, and representative textbooks. The first reviewer (ZLC) conducted electronic searches for the selected terms in these sources and extracted definitions into a data extraction form (Excel). When searching for definitions, we also searched for slight variations of the words.

### Assessment of the agreement of definitions

To evaluate the level of agreement among the retrieved definitions, a three-category ad hoc assessment scale was delineated: (1) agree meant that the definitions were similar and the essential understanding was present in each one; (2) partially agree meant that variations in the definitions were noted and one or more definitions did not convey similar information; and (3) do not agree meant that there was a fundamental difference in definitions.

The two senior pathologists (MDSL, VAW) independently reviewed the definitions and assessed the level of agreement.

### Analysis of results

A descriptive analysis was performed, specifying (1) number of sources defining the identified terms; (2) number of times a term was considered “defined,” “described, not defined,” and “not found”; and (3) number of agreements, partial agreements, and disagreements across the sources as assessed by the ad hoc developed scale.

## Results

Table 1 lists the 26 terms we identified and the reasons we considered them potentially misleading.

### Sources

We identified 16 relevant sources to search for definitions: seven institutions/organizations [16–22], three dictionaries [11–13], four websites [23–26], and two textbooks [27, 28]. The sources with their summarized definitions of terms are provided in Supplementary material.

We found no definitions for 4/26 (15%) terms: pseudoinvasion, osteoclast-like, serrated dysplasia, and malignant degeneration. Definitions of 3/26 (11.5%) terms, adenoma-like, cystic degeneration, and degenerative nuclear atypia, were only found in one source each and hence, could not be compared. Two of 26 (7.6%) terms, rhabdoid and carcinoid, were defined by the greatest number [10] of sources. Slight variations were noted between sources. In 27 instances, sources used terms and/or provided descriptions but did not specifically define them.

No single source provided definitions for all terms. Two sources [16, 18] had no definitions for any term. Pathology outlines [25] defined the most terms: 16 (61.5%), followed by Dorland’s Medical Dictionary [13]: 14 (53.8%). Generally, organizations defined fewer terms than the dictionaries, websites, and textbooks. From a total of 416 possible definitions (26 terms in 16 sources), we found 111 (26.7%).

### Agreement of definitions

Table 2 displays the sources that had definitions or descriptions only and the extent of agreement between these as assessed by two senior pathologists. Of the 26 identified terms, only 19 (73.1%) could be assessed as 7 (26.9%) had none or only one definition.

**Table 2:**
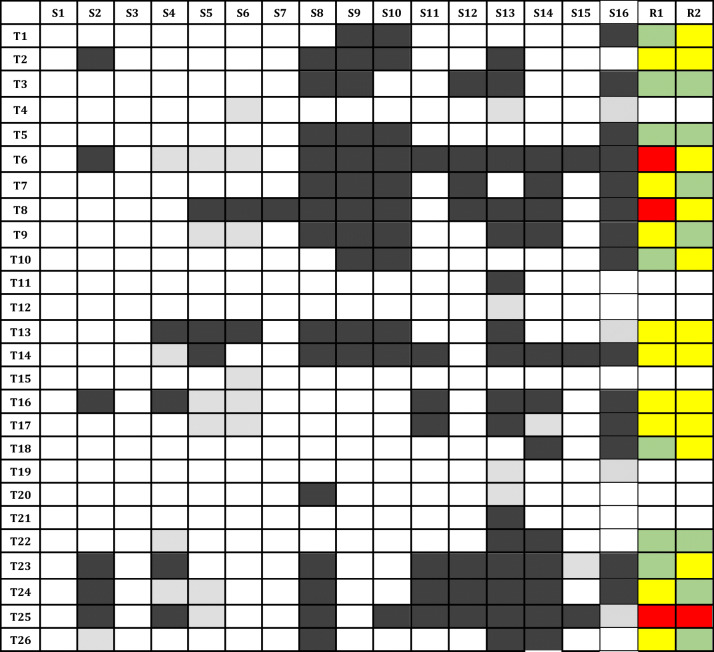
Illustration of the status of definitions found in sources and the ad hoc assessment of agreement

*T* terms; listed according to the sequence in Table 1; *S* sources; S1: World Health Organisation; S2: ICD-11; S3: European Society of Pathology; S4: College of American Pathologists; S5: Royal College of Pathologists; S6: Royal College of Pathologists of Australasia; S7: International Collaboration on Cancer Reporting; S8: Dorland’s medical dictionary; S9: Oxford English Dictionary; S10: Merriam-Webster Dictionary; S11: National Institutes of Health National Cancer Institute; S12: Medical Subject Headings; S13: PathologyOutlines; S14: Robbins and Cotran, Professional, 9^th^ Edition; S15: Schottenfeld and Fraumeni, Cancer Epidemiology and Prevention, 4^th^ Edition; S16: Wikipedia; *R* reviewers Status of definition: defined  Status of definition: described, not defined  Status of definition: no definition or description found  Agreement between definitions as assessed by reviewers: agree  Agreement between definitions as assessed by reviewers: partially agree  Agreement between definitions as assessed by reviewers: do not agree

The reviewers assessed that definitions agreed or partially agreed for 16 (84.2%) of the 19 terms that had two or more definitions (see Table 2). For one term, both reviewers felt that the definitions did not agree. This term was Paget disease (T25) in which not all definitions included both Paget disease of breast and Paget disease of bone, and one definition of Paget disease of breast said that malignant cells invaded the dermis. For two terms, the reviewers could not reach consensus on whether the definitions agreed. These were the terms of carcinoid (T6) and rhabdoid (T8) in which R1 felt that definitions were wrong and did not agree, while R2 felt that they partially agreed.

## Discussion

There is little written in the medical literature about the potential for medical terminology to be confusing or misleading. The terminology of a specific subspecialty may be well known to that particular group, but opaque to outsiders and new learners or used differently in other settings. Terminology usage in pathology is no different and many can remember the struggle as medical students to comprehend the plethora of unfamiliar terms. Many in the medical field might also be surprised at the subjectivity of anatomical pathology where diagnosis rests on visual impressions that have interpretive latitude. Terminology usage is also subjective and may be a matter of preference and style. Lack of a consensus and unfamiliarity with a definition are other reasons for questionable usage [7].

A definition is the starting point for clear terminology and the reason why each section of a WHO Blue Book begins with a concise definition of the tumor entity. This is particularly important for a classification that is used internationally, and which needs to consider cultural, idiomatic, and psychological aspects influencing comprehension. We found that concise, easy to understand definitions for pathologic terms were frequently lacking in sources commonly consulted by specialists. Terms were often described or used in the sources, without providing an actual definition. We considered that the finding of only 111 (26.7%) of 416 possible definitions (if all sources had provided definitions for all terms) seems low for a specialized field that underpins the diagnosis and treatment of cancer. However, there is nothing with which to compare this number.

Where present, definitions mainly agreed with each other: for 16 (84.2%) of 19 terms. This level of agreement does not mean that definitions were correct as they probably had not been decided by consensus, but do suggest that if defined, terms are consistently used and variations in definitions are not causes of confusion. However, in one instance, both reviewers agreed that the definitions did not agree with each other, and for two terms, one reviewer felt the definitions did not agree and the second reviewer felt that they only partially agreed. We acknowledge that it is actually difficult to determine the precise cutoff between “partially agree” and “do not agree” but this lack of agreement between the reviewers does indicate a problem with the definitions found in the sources.

We included the informal source Wikipedia [26] and found that it provided more definitions than most of the other consulted sources. However, we do not condone its use because of its lack of oversight and proper editorial review process.

We showed that sources commonly consulted by a wide variety of investigators, many non-native English speakers do not provide definitions for terms that might be used misleadingly. We used textbooks, websites of pathology and cancer organizations, and medical dictionaries in an attempt to cover a wide array of sources that might be consulted by different searchers but did not attempt to be exhaustive. We are particularly concerned about early career professionals, who need access to readily comprehensible definitions to avoid misunderstandings later in their careers.

We are aware that the list of potentially misleading terms was self-selected, but by using a consensus method among the WG, possible biases due to personal experiences were minimized. We focused on commonly used sources that might be consulted by learners or those outside the pathology field to provide a realistic picture of the status of definitions. There may also have been limitations in the searches due to the search engines of the websites.

We realize that judging the level of agreement between definitions is a subjective exercise, but by using a standardized assessment scale that we developed and by performing independent evaluations we sought to diminish bias as much as possible. We did not assess the usage of a particular term in its context since this was beyond the scope of this preliminary work but is considered for future research.

This investigation highlights the need for provision of definitions for terms used in the field of pathology and tumor classification to lessen subjectivity and improve the clarity of pathological diagnoses. This could be done by developing consensus definitions for terminology and a single authoritative source that can be consulted by a wide range of users.

## Supplementary Information

ESM 1 (DOCX 75 kb)
