# Correction to: Misleading terminology in pathology: Lack of definitions hampers communication

**DOI:** 10.1007/s00428-021-03101-w

**Published:** 2021-04-22

**Authors:** Zi Long Chow, Blanca Iciar Indave, Menaka Dilani Samarawickrema Lokuhetty, Atsushi Ochiai, Ian A. Cree, Valerie A. White

**Affiliations:** 1 WHO/IARC Classification of Tumours, International Agency for Research On Cancer (IARC), 150 Cours Albert Thomas, 69372 CEDEX 08 Lyon, France; 2 School of Medicine, University of Tasmania, 41 Charles St, Launceston, TAS 7250 Australia; 3 National Cancer Centre, Kashiwa, Japan

## Correction to: Virchows Archiv 10.1007/s00428-021-03069-7

The article “Misleading terminology in pathology: lack of definitions hampers communication”, written by Zi Long Chow, Blanca Iciar Indave, Menaka Dilani Samarawickrema Lokuhetty, Atsushi Ochiai, Ian A. Cree and Valerie A. With the author(s)’ decision to opt for Open Choice the copyright of the article changed on 26 March 2021 to © World Health Organization 2021 and the article is forthwith distributed under the terms of the Creative Commons Attribution IGO License http://creativecommons.org/licenses/by/3.0/igo/legalcode), which permits unrestricted use, distribution and reproduction in any medium, provided you give appropriate credit to the original author(s) and the source, provide a link to the Creative Commons license, and indicate if changes were made. In any reproduction of this article there should not be any suggestion that WHO or this article endorse any specific Organization or products. The use of the WHO logo is not permitted. This notice should be preserved along with the article’s original URL.

World Health Organization 2021 This is an open access article distributed under the terms of the Creative Commons Attribution IGO License (http://creativecommons.org/licenses/by/3.0/igo/legalcode), which permits unrestricted use, distribution and reproduction in any medium, provided you give appropriate credit to the original author(s) and the source, provide a link to the Creative Commons license, and indicate if changes were made. In any reproduction of this article there should not be any suggestion that WHO or this article endorse any specific Organization or products. The use of the WHO logo is not permitted. This notice should be preserved along with the article’s original URL.

