# Supplementary material for: Misleading terminology in pathology: lack of definitions hampers communication
Source: Virchows Arch. 2021 Apr 2;479(2):425–30. doi: 10.1007/s00428-021-03069-7 (PMC8364519; doi:10.1007/s00428-021-03069-7)
Supplement: Supplementary file 1 — (DOCX 75 kb) [file 428_2021_3069_MOESM1_ESM.docx]

**Supplementary Table 1: Retrieved definitions for terms with the prefix *pseudo*.**

Please click on the links to be redirected to the full definition.

| **Term** | **World Health Organization** | **ICD-11** | **European Society of Pathology** | **College of American Pathologists** | **Royal College of Pathologists** | **Royal College of Pathologists of Australasia** | **International Collaboration on Cancer Reporting** | **Dorland's medical dictionary** | **Oxford English Dictionary** | **Merriam-Webster Dictionary** | **National Institutes of Health National Cancer Institute** | **Medical Subject Headings** | **PathologyOutlines.com** | **Robbins and Cotran Pathologic Basis of Disease, Professional Edition, 9th Edition** | **Schottenfeld and Fraumeni, Cancer Epidemiology and Prevention, 4th Edition** | **Wikipedia** |
| --- | --- | --- | --- | --- | --- | --- | --- | --- | --- | --- | --- | --- | --- | --- | --- | --- |
| **Pseudo** |  |  |  |  |  |  |  |  | [False or spurious; pretended, fake; so-called.](https://www.oed.com/view/Entry/153742?rskey=bQ3cRo&result=2&isAdvanced=false#eid) | [Being apparently rather than actually as stated](https://www.merriam-webster.com/dictionary/pseudo) |  |  |  |  |  | [Used to mark something that superficially appears to be one thing, but is something else.](https://es.wikipedia.org/wiki/Pseudo) |
| **Pseudotumour** |  | [Any condition by determinants acquired after birth, … leading to tumour-like conditions of the spleen.](https://icd.who.int/browse11/l-m/en#/http://id.who.int/icd/entity/142317144) |  |  |  |  |  | [An enlargement that resembles a tumour; …](https://www.dorlandsonline.com/dorland/definition?id=41853&searchterm=pseudotumor) | [A mass or other lesion that gives rise to signs or symptoms suggestive of a neoplasm …](https://www.oed.com/view/Entry/238284?redirectedFrom=Pseudotumour#eid) | [(Medical) an abnormality (as a temporary swelling) that resembles a tumour.](https://www.merriam-webster.com/medical/pseudotumor) |  |  | [Category used to designate a variety of benign proliferative lesions forming a lung mass…](https://www.pathologyoutlines.com/topic/lungtumorinflammatorypseudotumor.html) |  |  |  |
| **Pseudolymphoma** |  |  |  |  |  |  |  | [Any group of disorders having a benign course but exhibiting clinical and histological features suggestive of malignant lymphoma…](https://www.dorlandsonline.com/dorland/definition?id=41749&searchterm=pseudolymphoma) | [A mass of infiltrate of lymphoid cells that has some (histopathological) features resembling lymphoma but is not malignant.](https://www.oed.com/view/Entry/238235?redirectedFrom=Pseudolymphoma#eid) |  |  | [A group of disorders having a benign course but exhibiting clinical and histological features suggestive of malignant lymphoma](https://www.ncbi.nlm.nih.gov/mesh/?term=pseudolymphoma). | [Called lymphoma-like lesion; a form of chronic cervicitis. …](https://www.pathologyoutlines.com/topic/cervixpseudolymphoma.html) |  |  | [Pseudolymphoma is a benign lymphocytic infiltrate that resembles cutaneous lymphoma histologically, clinically, or both.](https://en.wikipedia.org/wiki/Pseudolymphoma) |
| **Pseudoinvasion/epithelial misplacement** |  |  |  |  |  | Described only. |  |  |  |  |  |  | Described only. |  |  | [Infrequently, the epithelium is misplacement into the submucosa…](https://en.wikipedia.org/wiki/Hyperplastic_polyp#Epithelial_misplacement) |

**Footnote:** GREEN cells mean definition provided RED cells mean no definition provided, only a description YELLOW cells mean no definition found.

Both Robbins and Cotran, Pathologic Basis of Disease, Professional Edition, 9^th^ Edition, and Schottenfeld and Fraumeni, Cancer Epidemiology and Prevention, 4^th^ Edition are paper-based text.

Wikipedia (highlighted in grey) has been included as one of the sources because of current reality. The authors recognise that Wikipedia does not have the equivalent level of credibility as the other sources.

**Supplementary Table 2: Retrieved definitions for terms with the suffix *-oid*.**

Please click on the links to be redirected to the full definition.

| **Term** | **World Health Organization** | **ICD-11** | **European Society of Pathology** | **College of American Pathologists** | **Royal College of Pathologists** | **Royal College of Pathologists of Australasia** | **International Collaboration on Cancer Reporting** | **Dorland's medical dictionary** | **Oxford English Dictionary** | **Merriam-Webster Dictionary** | **National Institutes of Health National Cancer Institute** | **Medical Subject Headings** | **PathologyOutlines.com** | **Robbins and Cotran Pathologic Basis of Disease, Professional Edition, 9th Edition** | **Schottenfeld and Fraumeni, Cancer Epidemiology and Prevention, 4th Edition** | **Wikipedia** |
| --- | --- | --- | --- | --- | --- | --- | --- | --- | --- | --- | --- | --- | --- | --- | --- | --- |
| **(-oid)** |  |  |  |  |  |  |  | [A word termination denoting resemblance to the thing specified by the stem to which it is affixed, as ovoid.](https://www.dorlandsonline.com/dorland/definition?id=100074404&searchterm=-oid) | [Forming adjectives with the sense ‘...resembling, allied to’, and nouns with the …](https://www.oed.com/view/Entry/130874?redirectedFrom=oid#eid) | [Noun suffix: something resembling a (specified) object or having a (specified) quality; …](https://www.merriam-webster.com/dictionary/-oid) |  |  |  |  |  | [Of similar form to, but not the same as. Having the likeness of…](https://en.wiktionary.org/wiki/-oid) |
| **Carcinoid** |  | [Malignant neoplasms with neuroendocrine differentiation…](https://icd.who.int/browse11/l-m/en#/http://id.who.int/icd/entity/1590340268) |  | Described only. | Described only. | Described only. |  | [Carcinoid tumour, which is defined as a small, slow-growing neuroendocrine tumour arising from enterochromaffin cells and occurring most often in the gastrointestinal tract and lung](https://www.dorlandsonline.com/dorland/definition?id=115718) | [(1) of the nature of or resembling cancer; (2) noun: a tumour derived from argentaffin neuroendocrine cells, which is found most commonly in the gastrointestinal tract](https://www.oed.com/view/Entry/27821?redirectedFrom=Carcinoid#eid) | [A benign or malignant tumour arising especially from the mucosa of the gastrointestinal tract](https://www.merriam-webster.com/dictionary/carcinoid) | [Carcinoid tumour: a slow-growing type of tumour usually found in the gastrointestinal system (most often in the small intestine and rectum), and sometimes in the lungs or other sites.](https://www.cancer.gov/publications/dictionaries/cancer-terms/def/carcinoid-tumor) | [It is now established that these tumours are of neuroendocrine origin and derive from a primitive stem cell](https://meshb.nlm.nih.gov/record/ui?ui=D002276). | [Synonym: neuroendocrine tumour (low grade) = carcinoid tumour, atypical carcinoid tumour](https://www.pathologyoutlines.com/topic/esophaguscarcinoid.html). | Carcinoid tumours arise from the diffuse components of the endocrine system and are now properly referred to as well-differentiated neuroendocrine tumours. (page 1562) | Carcinoid tumours ...are neuroendocrine tumours … capable of producing serotonin… (page 672) | [A carcinoid (also carcinoid tumour) is a slow-growing type of neuroendocrine tumour originating in the cells of the neuroendocrine system.](https://en.wikipedia.org/wiki/Carcinoid) |
| **Epithelioid** |  |  |  |  |  |  |  | [Resembling epithelium.](https://www.dorlandsonline.com/dorland/definition?id=16978&searchterm=epithelioid) | [Resembling epithelium.](https://www.oed.com/view/Entry/63597?redirectedFrom=Epithelioid#eid) | [Resembling epithelium.](https://www.merriam-webster.com/dictionary/epithelioid) |  | Epithelioid cells: [characteristic cells of granulomatous hypersensitivity](https://meshb.nlm.nih.gov/record/ui?ui=D015622). |  | Epithelioid cells are spherical and have greater cytologic atypicality. (page 2711) |  | [Epithelioid histiocytes (Epithelioid cells) are activated macrophages resembling epithelial cells.](https://en.wikipedia.org/wiki/Epithelioid) |
| **Rhabdoid** |  |  |  |  | [Rhabdoid morphology refers to the presence of epithelioid cells with …](https://www.rcpath.org/uploads/assets/32952412-75e2-4008-83cd204863373aa1/G037-Dataset-for-histopathological-reporting-of-adult-renal-parenchyma-neoplasms.pdf) | [Tumours showing this phenotype resemble rhabdoid cells having bulky eosinophilic cytoplasm and...](https://www.rcpa.edu.au/Library/Practising-Pathology/Structured-Pathology-Reporting-of-Cancer/Docs/P11_S3_04) | [Tumours showing this phenotype resemble rhabdoid cells having bulky eosinophilic cytoplasm and...](http://www.iccr-cancer.org/datasets/docs/iccr-renalbx-rhabdoid) | [Resembling a rod; rod-shaped](https://www.dorlandsonline.com/dorland/definition?id=43661&searchterm=rhabdoid) | [Characteristic of or resembling a rhabdomyosarcoma.](https://www.oed.com/view/Entry/165092?redirectedFrom=Rhabdoid+#eid) | [Shaped like a rod or rhaboidal.](https://www.merriam-webster.com/dictionary/rhabdoid) |  | [Histopathologically, it resembles rhabdomyosarcoma but the tumour cells are not of myogenic origin.](https://meshb.nlm.nih.gov/record/ui?ui=D018335) | [Rhabdoid: dyscohesive cells with abundant eosinophilic cytoplasm](http://www.pathologyoutlines.com/topic/pleuramesotheliomaepithelioid.html) ... | …includes rhabdoid cells, resembling those of a rhabdomyosarcoma… (page 2671) |  | [The term rhabdoid was used due to its similarity with rhabdomyosarcoma](https://en.wikipedia.org/wiki/Atypical_teratoid_rhabdoid_tumor). |
| **Pagetoid** |  |  |  |  | Described only. | Described only. |  | [Resembling or characteristic of Paget disease](https://www.dorlandsonline.com/dorland/definition?id=100077415&searchterm=pagetoid) | [Resembling or characteristic of (that of) Paget's disease of the skin.](https://www.oed.com/view/Entry/263271?rskey=fDamY6&result=1#eid10511979) | [Belonging to or typical of Paget's disease](https://www.merriam-webster.com/medical/pagetoid) |  |  | ["Pagetoid spread" is characteristic spread of malignant cells ...](https://www.pathologyoutlines.com/topic/breastmalignantlcis.html) | Urothelium and Breast (page 1963 and 2157): Pagetoid spread, ...neoplastic cells between the basement membrane and overlying luminal cells. |  | [The term Pagetoid (Paget-like) is derived from the Extramammary Paget's disease, wherein the large tumour cells…](https://en.wikipedia.org/wiki/Pagetoid) |

**Footnote:** GREEN cells mean definition provided RED cells mean no definition provided, only a description YELLOW cells mean no definition found.

Both Robbins and Cotran, Pathologic Basis of Disease, Professional Edition, 9^th^ Edition, and Schottenfeld and Fraumeni, Cancer Epidemiology and Prevention, 4^th^ Edition are paper-based text.

Wikipedia (highlighted in grey) has been included as one of the sources because of current reality. The authors recognise that Wikipedia does not have the equivalent level of credibility as the other sources.

**Supplementary Table 3: Retrieved definitions for the term *microinvasion* and terms with the suffix *-like*.**

Please click on the links to be redirected to the full definition.

| **Term** | **World Health Organization** | **ICD-11** | **European Society of Pathology** | **College of American Pathologists** | **Royal College of Pathologists** | **Royal College of Pathologists of Australasia** | **International Collaboration on Cancer Reporting** | **Dorland's medical dictionary** | **Oxford English Dictionary** | **Merriam-Webster Dictionary** | **National Institutes of Health National Cancer Institute** | **Medical Subject Headings** | **PathologyOutlines.com** | **Robbins and Cotran Pathologic Basis of Disease, Professional Edition, 9th Edition** | **Schottenfeld and Fraumeni, Cancer Epidemiology and Prevention, 4th Edition** | **Wikipedia** |
| --- | --- | --- | --- | --- | --- | --- | --- | --- | --- | --- | --- | --- | --- | --- | --- | --- |
| **Micro-invasion** |  |  |  | [Defined by AJCC as invasion measuring 1mm or less in size](https://documents.cap.org/protocols/cp-breast-invasive-resection-19-4301.pdf). | [Less than 1mm across in largest diameter, should be categorised under invasive if there is no associated DCIS](https://www.rcpath.org/uploads/assets/7763be1c-d330-40e8-95d08f955752792a/G148_BreastDataset-hires-Jun16.pdf)… | [Invasive tumours measuring 1mm or less are classified as microinvasive (pT1mic).](https://www.rcpa.edu.au/getattachment/9c857cb6-6878-4004-bf8a-37761873cf13/Protocol-invasive-breast-cancer.aspx) |  | [Microscopic extension of malignant cells into adjacent tissue in carcinoma in situ.](https://www.dorlandsonline.com/dorland/definition?id=100066377&searchterm=microinvasion) | [Of a neoplasm: invasive at a microscopic level.](https://www.oed.com/view/Entry/276415?redirectedFrom=microinvasive#eid) | [Of, relating to, or characterised by a very slight invasion into adjacent tissues by malignant cells of a carcinoma in situ](https://www.merriam-webster.com/medical/microinvasion) |  |  | Defined in [ovarian](https://www.pathologyoutlines.com/topic/ovarytumormicroinvasive.html), [bladder and ureters](http://www.pathologyoutlines.com/topic/bladdercis.html), and [breast](https://www.pathologyoutlines.com/topic/breastmalignantmicroinvasion.html) pages. |  |  | Described only. |
| **(-like)** |  |  |  |  |  |  |  |  | [Nouns: Forming adjectives with the sense ‘similar to or of the nature of —’,](https://www.oed.com/view/Entry/108305) | [Resembling or characteristic of](https://www.merriam-webster.com/dictionary/like) |  |  |  |  |  | [Having some of the characteristics of (used to form adjectives from nouns).](https://en.wiktionary.org/wiki/-like) |
| **Adenoma-like** |  |  |  |  |  |  |  |  |  |  |  |  | [An invasive carcinoma with architectural and cytologic features resembling villous adenoma](https://www.pathologyoutlines.com/topic/colontumortvadenoma.html). |  |  |  |
| **Osteoclast-like** |  |  |  |  |  |  |  |  |  |  |  |  | Described only. |  |  |  |

**Footnote:** GREEN cells mean definition provided RED cells mean no definition provided, only a description YELLOW cells mean no definition found.

Both Robbins and Cotran, Pathologic Basis of Disease, Professional Edition, 9^th^ Edition, and Schottenfeld and Fraumeni, Cancer Epidemiology and Prevention, 4^th^ Edition are paper-based text.

Wikipedia (highlighted in grey) has been included as one of the sources because of current reality. The authors recognise that Wikipedia does not have the equivalent level of credibility as the other sources.

**Supplementary Table 4: Retrieved definitions for the term d*ysplasia*.**

Please click on the links to be redirected to the full definition.

| **Term** | **World Health Organization** | **ICD-11** | **European Society of Pathology** | **College of American Pathologists** | **Royal College of Pathologists** | **Royal College of Pathologists of Australasia** | **International Collaboration on Cancer Reporting** | **Dorland's medical dictionary** | **Oxford English Dictionary** | **Merriam-Webster Dictionary** | **National Institutes of Health National Cancer Institute** | **Medical Subject Headings** | **PathologyOutlines.com** | **Robbins and Cotran Pathologic Basis of Disease, Professional Edition, 9th Edition** | **Schottenfeld and Fraumeni, Cancer Epidemiology and Prevention, 4th Edition** | **Wikipedia** |
| --- | --- | --- | --- | --- | --- | --- | --- | --- | --- | --- | --- | --- | --- | --- | --- | --- |
| **Dysplasia** |  |  |  | Described only. | [Intraepithelial neoplasia](https://www.rcpath.org/uploads/assets/cbae24ce-220c-4d9f-95c5b83dd1f0ad2b/Dataset-for-histopathology-reporting-of-liver-resection-specimens-and-liver-biopsies-for-primary-and-metastatic-carcinoma-2nd-edition.pdf) |  |  | [(1) Abnormality of development OR (2) in pathology, alteration in size, shape, and organisation of adult cells.](https://www.dorlandsonline.com/dorland/definition?id=100033210&searchterm=dysplasia) | [Pathology. abnormal growth or growth of tissues, cells, etc](https://www.oed.com/view/Entry/58901?redirectedFrom=dysplasia#eid) | [Abnormal growth of or development (as of organs or cells).](https://www.merriam-webster.com/dictionary/dysplasia) | [Cells that look abnormal under a microscope but are not cancer.](https://www.cancer.gov/publications/dictionaries/cancer-terms/def/dysplasia) |  | [Dysplasia, a premalignant condition, refers to](https://www.pathologyoutlines.com/topic/oralcavitydysplasia.html) ... | "Disordered growth"… (page 531). | Epithelial intraepithelial neoplasia terminology is conceptualized as a universal nomenclature for carcinoma precursor… (page 22) | [An abnormal growth or…](https://en.wikipedia.org/wiki/Dysplasia) |
| **Serrated dysplasia** |  |  |  |  |  | Described only. |  |  |  |  |  |  |  |  |  |  |
| **High-grade dysplasia** |  | [...this condition is characterised by premalignant transformation and severe dysplasia of the cervix…](https://icd.who.int/browse11/l-m/en#/http%3a%2f%2fid.who.int%2ficd%2fentity%2f1656189375) |  | [… cancer cells confined within the glandular basement membrane (high-grade dysplasia)](https://documents.cap.org/protocols/cp-ampulla-17protocol-4000.pdf) | Described only | Described only |  |  |  |  | [An area of abnormal cells that forms on the surface of certain organs, such as the cervix, vagina, vulva, anus and oesophagus.](https://www.cancer.gov/publications/dictionaries/cancer-terms/def/high-grade-squamous-intraepithelial-lesion) |  | [Severe cytologic atypia, glandular cribiforming or full thickness nuclear stratification](https://www.pathologyoutlines.com/topic/stomachdysplasia.html). | High-grade dysplasia is "characterized by more severe cytologic atypia and … (page 1554) |  | [A localized phenomenon, with no potential for metastasis unless it progresses into cancer](https://en.wikipedia.org/wiki/Carcinoma_in_situ). |
| **Low-grade dysplasia** |  |  |  |  | Described only. | Described only. |  |  |  |  | [Slightly abnormal cells are found on the surface of the cervix.](https://www.cancer.gov/publications/dictionaries/cancer-terms/def/low-grade-squamous-intraepithelial-lesion) |  | [Nuclear enlargement, hyperchromatism, stratification](https://www.pathologyoutlines.com/topic/stomachdysplasia.html). | Described only. |  | [LSIL usually indicated mild dysplasia (CIN 1).](https://en.wikipedia.org/wiki/Bethesda_system) |
| **Dysplastic epithelium** |  |  |  |  |  |  |  |  |  |  |  |  |  | Dysplastic epithelial cells with an increased N:C… (page 1633) |  | [Epithelial dysplasia ...is the sum of various disturbances of epithelial proliferation and differentiation](https://en.wikipedia.org/wiki/Epithelial_dysplasia)… |

**Footnote:** GREEN cells mean definition provided RED cells mean no definition provided, only a description YELLOW cells mean no definition found.

Both Robbins and Cotran, Pathologic Basis of Disease, Professional Edition, 9^th^ Edition, and Schottenfeld and Fraumeni, Cancer Epidemiology and Prevention, 4^th^ Edition are paper-based text.

Wikipedia (highlighted in grey) has been included as one of the sources because of current reality. The authors recognise that Wikipedia does not have the equivalent level of credibility as the other sources.

**Supplementary Table 5: Retrieved definitions for the term *degeneration*.**

Please click on the links to be redirected to the full definition.

| **Term** | **World Health Organization** | **ICD-11** | **European Society of Pathology** | **College of American Pathologists** | **Royal College of Pathologists** | **Royal College of Pathologists of Australasia** | **International Collaboration on Cancer Reporting** | **Dorland's medical dictionary** | **Oxford English Dictionary** | **Merriam-Webster Dictionary** | **National Institutes of Health National Cancer Institute** | **Medical Subject Headings** | **PathologyOutlines.com** | **Robbins and Cotran Pathologic Basis of Disease, Professional Edition, 9th Edition** | **Schottenfeld and Fraumeni, Cancer Epidemiology and Prevention, 4th Edition** | **Wikipedia** |
| --- | --- | --- | --- | --- | --- | --- | --- | --- | --- | --- | --- | --- | --- | --- | --- | --- |
| **Malignant degeneration** |  |  |  |  |  |  |  |  |  |  |  |  | Described only. |  |  | Described only. |
| **Cystic degeneration** |  |  |  |  |  |  |  | [Degeneration with formation of cysts.](https://www.dorlandsonline.com/dorland/definition?id=68852&searchterm=cystic+degeneration) |  |  |  |  | Described only. |  |  |  |
| **Degenerative nuclear atypia** |  |  |  |  |  |  |  |  |  |  |  |  | [Degenerative nuclear atypia (ancient change) can show nuclear pleomorphism and occasionally nuclear inclusions](https://www.pathologyoutlines.com/topic/softtissueschwannoma.html). |  |  |  |
| **Degenerative changes** |  |  |  | Described only. |  |  |  |  |  |  |  |  | [Degenerative changes include hyalinized blood vessels](https://www.pathologyoutlines.com/topic/cnstumorpilocyticastrocytoma.html)… | Degenerative changes may include nuclear pleomorphism… (page 2531) |  |  |

**Footnote:** GREEN cells mean definition provided RED cells mean no definition provided, only a description YELLOW cells mean no definition found.

Both Robbins and Cotran, Pathologic Basis of Disease, Professional Edition, 9^th^ Edition, and Schottenfeld and Fraumeni, Cancer Epidemiology and Prevention, 4^th^ Edition are paper-based text.

Wikipedia (highlighted in grey) has been included as one of the sources because of current reality. The authors recognise that Wikipedia does not have the equivalent level of credibility as the other sources.

**Supplementary Table 6: Retrieved definitions for selected *eponyms* and *Latinate terms*.**

Please click on the links to be redirected to the full definition.

| **Term** | **World Health Organization** | **ICD-11** | **European Society of Pathology** | **College of American Pathologists** | **Royal College of Pathologists** | **Royal College of Pathologists of Australasia** | **International Collaboration on Cancer Reporting** | **Dorland's medical dictionary** | **Oxford English Dictionary** | **Merriam-Webster Dictionary** | **National Institutes of Health National Cancer Institute** | **Medical Subject Headings** | **PathologyOutlines.com** | **Robbins and Cotran Pathologic Basis of Disease, Professional Edition, 9th Edition** | **Schottenfeld and Fraumeni, Cancer Epidemiology and Prevention, 4th Edition** | **Wikipedia** |
| --- | --- | --- | --- | --- | --- | --- | --- | --- | --- | --- | --- | --- | --- | --- | --- | --- |
| **Barrett Oesophagus** |  | [Columnar metaplastic epithelium of the oesophagus, unspecified](https://icd.who.int/browse11/l-m/en#/http://id.who.int/icd/entity/1442921818/mms/unspecified) |  | [Intestinal metaplasia](https://documents.cap.org/protocols/cp-esophagus-17protocol-4000.pdf) |  |  |  | [Peptic ulcer of the lower oesophagus, often with stricture, due to the presence of columnar-lined epithelium in the oesophagus](https://www.dorlandsonline.com/dorland/definition?id=100103878&searchterm=Barrett%20syndrome)… |  |  | [A condition in which the cells lining the lower part of the oesophagus have changed or been replaced with abnormal cells that could lead to cancer of the oesophagus](https://www.cancer.gov/publications/dictionaries/cancer-terms/def/barrett-esophagus). | [A condition with damage to the lining of the lower exophage resulting from chronic acid reflux](https://meshb.nlm.nih.gov/record/ui?ui=D001471) ... | [Distal squamous mucosa is replaced by metaplastic specialised (intestinalized columnar) epithelium](http://www.pathologyoutlines.com/topic/esophagusBarrettsgeneral.html)… | Complication of chronic GORD that is characterised by intestinal metaplasia within the oesophageal squamous mucosa. (page 1524) | Described only. | [A condition in which there is metaplastic change in the mucosal cells lining the lower portion of the oesophagus](https://en.wikipedia.org/wiki/Barrett%27s_esophagus)… |
| **Langerhans cell histiocytosis** |  | [A neoplastic proliferation of Langerhans cells which contain Birbceck granules by ultrastructural examination](https://icd.who.int/browse11/l-m/en#/http://id.who.int/icd/entity/216625985). |  | Described only. | Described only. |  |  | …[characterized by proliferation of Langerhans cells, affecting children more often than adults](https://www.dorlandsonline.com/dorland/definition?id=200018660&searchterm=Langerhans+cell+histiocytosis)… |  |  | [A group of rare disorders in which too many Langerhans cells grow in certain tissues and organs](https://www.cancer.gov/publications/dictionaries/cancer-terms/def/lch)... | [A group of disorders resulting from the abnormal proliferation of the tissue infiltration by Langerhans cells](https://www.ncbi.nlm.nih.gov/mesh/68006646)… | Clonal proliferation of Langerhans cells, found in: [lymph nodes](http://www.pathologyoutlines.com/topic/lymphnodesLCH.html), [skin nonmelanocytic tumour](http://www.pathologyoutlines.com/topic/skintumornonmelanocyticLCH.html), [lung tumour](http://www.pathologyoutlines.com/topic/lungtumorLCH.html), [mandible and maxilla](http://www.pathologyoutlines.com/topic/mandiblemaxillaLCH.html), [CNS tumour](https://www.pathologyoutlines.com/topic/cnstumorLCH.html), [mediastinum](http://www.pathologyoutlines.com/topic/mediastinumLCH.html), [liver](https://www.pathologyoutlines.com/topic/livertumorlangerhanscell.html), [thyroid](http://www.pathologyoutlines.com/topic/thyroidlch.html), [spleen](http://www.pathologyoutlines.com/topic/spleenLCH.html), [stomach](https://www.pathologyoutlines.com/topic/stomachLCH.html), and [ear](http://www.pathologyoutlines.com/topic/earLCH.html). | …a spectrum of proliferations of a special type of immature dendritic cell called Langerhans cell. (page 1243 – 1245) |  | [Langerhans cell histiocytosis is a rare cancer involving clonal proliferation of Langerhans cells](https://en.wikipedia.org/wiki/Langerhans_cell_histiocytosis)… |
| **Paget disease** |  | Diverse definitions available for [mammary](https://icd.who.int/browse11/l-m/en#/http://id.who.int/icd/entity/1295910447), [extramammary](https://icd.who.int/browse11/l-m/en#/http://id.who.int/icd/entity/1913185137) and [bone](https://icd.who.int/browse11/l-m/en#/http%3a%2f%2fid.who.int%2ficd%2fentity%2f1653656681). |  | [When DCIS involves nipple skin only, without underling invasive carcinoma or DCIS.](https://documents.cap.org/protocols/cp-breast-dcis-biopsy-19-1000.docx) | Described only. |  |  | [Intraductal carcinoma of the breast extending to involve the nipple and areola](https://www.dorlandsonline.com/dorland/definition?id=100031173&searchterm=Paget+disease)... |  | [Breast](https://www.merriam-webster.com/dictionary/Paget%27s%20disease): ...rare ...initially manifested as a scaly red rash on the nipple and areola. [Bone](https://www.merriam-webster.com/dictionary/Paget%27s%20disease): a chronic disease that is characterized by one or more enlarged, weak bones … | [Bone](https://www.cancer.gov/publications/dictionaries/cancer-terms/def/paget-disease-of-bone): a chronic condition in which both the breakdown and regrowth of bone are increased. [Nipple](https://www.cancer.gov/publications/dictionaries/cancer-terms/def/breast-carcinoma-in-situ): a condition in which abnormal cells are found in the nipple. | [Bone, mammary and extramammary](https://www.ncbi.nlm.nih.gov/mesh/?term=paget+disease) definition found. | Diverse definitions available for [mammary](https://www.pathologyoutlines.com/topic/breastmalignantpaget.html), extramammary ([anus](https://www.pathologyoutlines.com/topic/anuspagets.html?mobile=off) & [penis](https://www.pathologyoutlines.com/topic/penscrotumpenilepagets.html)) and [bone](https://www.pathologyoutlines.com/topic/bonepagets.html). | Found in page 2033 (extramammary), 2155 (breast), and 2408 (bone). | Bone: … characterized by highly exaggerated bone remodelling… (page 821) | Described only. |
| **Latinate terms: as in leiomyomatosis peritonealis disseminata** |  | Described only. |  |  |  |  |  | [Abdominal smooth muscle tumours… scattered throughout the peritoneal surfaces](https://www.dorlandsonline.com/dorland/definition?id=100058251&searchterm=leiomyomatosis+peritonealis+disseminata)… |  |  |  |  | Included a version of the blue book in search result, which defined it as "a benign entity in which numerous small nodules composed of smooth muscle are present in the peritoneal cavity". | A variant of leiomyomas, which present as multiple small peritoneal nodules and benign. (page 2075) |  |  |

**Footnote:** GREEN cells mean definition provided RED cells mean no definition provided, only a description YELLOW cells mean no definition found.

Both Robbins and Cotran, Pathologic Basis of Disease, Professional Edition, 9^th^ Edition, and Schottenfeld and Fraumeni, Cancer Epidemiology and Prevention, 4^th^ Edition are paper-based text.

Wikipedia (highlighted in grey) has been included as one of the sources because of current reality. The authors recognise that Wikipedia does not have the equivalent level of credibility as the other sources.
